# Antimicrobial Constituents from *Machaerium* Pers.: Inhibitory Activities and Synergism of Machaeriols and Machaeridiols against Methicillin-Resistant *Staphylococcus aureus*, Vancomycin-Resistant *Enterococcus faecium*, and Permeabilized Gram-Negative Pathogens

**DOI:** 10.3390/molecules25246000

**Published:** 2020-12-18

**Authors:** Ilias Muhammad, Melissa R. Jacob, Mohamed A. Ibrahim, Vijayasankar Raman, Mallika Kumarihamy, Mei Wang, Taha Al-Adhami, Charlotte Hind, Melanie Clifford, Bethany Martin, Jianping Zhao, J. Mark Sutton, Khondaker Miraz Rahman

**Affiliations:** 1 National Center for Natural Products Research, Research Institute of Pharmaceutical Sciences, School of Pharmacy, University of Mississippi, Oxford, MS 38677, USA; mellie2880@gmail.com (M.R.J.); mmibrahi@olemiss.edu (M.A.I.); vraman@olemiss.edu (V.R.); mkumarih@olemiss.edu (M.K.); meiwang@olemiss.edu (M.W.); jianping@olemiss.edu (J.Z.); 2 Natural Products Utilization Research Unit, Agricultural Research Service, U.S. Department of Agriculture, University of Mississippi, Oxford, MS 38677, USA; 3 Institute of Pharmaceutical Science, School of Cancer and Pharmaceutical Sciences, King’s College London, Franklin-Wilkins Building, 150 Stamford Street, London SE1 9NH, UK; taha.al-adhami@kcl.ac.uk; 4 National Infection Service, Public Health England, Manor Farm Road, Salisbury SP4 0JG, UK; Charlotte.Hind@phe.gov.uk (C.H.); Melanie.Clifford@phe.gov.uk (M.C.); Bethany.martin@phe.gov.uk (B.M.); mark.sutton@phe.gov.uk (J.M.S.)

**Keywords:** *Machaerium* Rimachi 12161, machaerifurogerol, 5-*epi*-machaerifurogerol, machaeriol A–C, machaeridiol A–C, isoflavonoid, MRSA, VRE, gram negative bacteria

## Abstract

Two new epimeric bibenzylated monoterpenes machaerifurogerol (**1a**) and 5-*epi*-machaerifurogerol (**1b**), and four known isoflavonoids (+)-vestitol (**2**), 7-*O*-methylvestitol (**3**), (+)-medicarpin (**4**), and 3,8-dihydroxy-9-methoxypterocarpan (**5**) were isolated from *Machaerium* Pers. This plant was previously assigned as *Machaerium multiflorum* Spruce, from which machaeriols A-D (**6**–**9**) and machaeridiols A-C (**10**–**12**) were reported, and all were then re-isolated, except the minor compound **9**, for a comprehensive antimicrobial activity evaluation. Structures of the isolated compounds were determined by full NMR and mass spectroscopic data. Among the isolated compounds, the mixture **10** + **11** was the most active with an MIC value of 1.25 μg/mL against methicillin-resistant *Staphylococcus aureus* (MRSA) strains BAA 1696, −1708, −1717, −33591, and vancomycin-resistant *Enterococcus faecium* (VRE 700221) and *E*. *faecalis* (VRE 51299) and vancomycin-sensitive *E. faecalis* (VSE 29212). Compounds **6**–**8** and **10**–**12** were found to be more potent against MRSA 1708, and **6**, **11**, and **12** against VRE 700221, than the drug control ciprofloxacin and vancomycin. A combination study using an in vitro Checkerboard method was carried out for machaeriols (**7** or **8**) and machaeridiols (**11** or **12**), which exhibited a strong synergistic activity of **12** + **8** (MIC 0.156 and 0.625 µg/mL), with >32- and >8-fold reduction of MIC’s, compared to **12**, against MRSA 1708 and −1717, respectively. In the presence of sub-inhibitory concentrations on polymyxin B nonapeptide (PMBN), compounds **10** + **11**, **11**, **12**, and **8** showed activity in the range of 0.5–8 µg/mL for two strains of *Acinetobacter baumannii*, 2–16 µg/mL against *Pseudomonas aeruginosa* PAO1, and 2 µg/mL against *Escherichia coli* NCTC 12923, but were inactive (MIC > 64 µg/mL) against the two isolates of *Klebsiella pneumoniae*.

## 1. Introduction

The genus *Machaerium* Pers. (Fabaceae) consists of approximately 130 species, which are primarily distributed in the tropical Americas [1]. It is a genus of shrubs or lianas and small to medium-sized trees occurring throughout Southern Mexico to Brazil and Northern Argentina and Peru. These species are indigenous to all climatic regions ranging from equatorial rainforests to the verges of dry and cold deserts [2,3,4]. Several species of this genus are used in traditional medicines are considered to have multiple medicinal properties. Generally, various plant parts of *Machaerium* are used as an antitussive, and the sap is used to cure aphthous ulcers of the mouth [4]. *M. floribundum* is used to treat diarrhea and menstrual cramps [4]. The presence of a wide array of secondary metabolites from *Machaerium*, including flavonoids, terpenoids, and oxygenated phenolic compounds, together with their bioactivities, was recently reviewed by Amen et al. (2015) [2].

Earlier studies on one of the *Machaerium* species (Manuel Rimachi, Y-12161), named *M. multiflorum* Spruce, yielded four unique (+)-*trans*-hexahydrodibenzopyrans (HHDBP), machaeriols A-D, and three 5,6-*seco*-HHDBPs, machaeridiols A-C [5,6]. An unprecedented structural similarity for the HHDBP nucleus was observed in machaeriol and hexahydrocannabinol, and the 5,6-*seco*-HHDBP nucleus in machaeridiol and dihydrocannabidiol. Since these are the first reports of novel phytocannabinoids from a higher plant other than *Cannabis*, a recollection of the plant material was necessary from the original source. Unfortunately, there is an absence of documentary evidence for the existence of the species *M. multiflorum*. This species name was not included in the regional Floras as well as in major online databases (i.e., the International Plant Names Index (http://www.ipni.org/index.html) and The Plant List (http://www.theplantlist.org). Therefore, it was assumed that the plant sample was misidentified and was given the name combination *M. multiflorum* Spruce in error. An investigation was carried out on the identity of the plant, and a re-examination of the voucher specimen (Rimachi # 12161) at the Missouri Botanical Garden (MBG) concluded that this species should be treated only as an unidentified species of *Machaerium* Pers., as determined by the collection information (Manuel Rimachi, Y. 12161) [7].

The significance of the chemistry and biological activity of these aralkyl class of phytocannabinoid-type compounds led to the re-examination of the *n*-hexane and DCM fractions of the stem bark EtOH extract of the original plant material [5,6], as well as previously unexamined root and leaf extracts, which showed significant enhancement of antimicrobial activity against the various strains of methicillin-resistant *Staphylococcus aureus* (MRSA) and vancomycin resistant *Enterococci* (VRE). MRSA and VRE represent two potential threats to human health. According to the Centers for Disease Control and Prevention (CDC), MRSA can cause serious health problems, such as bloodstream infections and pneumonia. CA-MRSA occurs with a higher incidence rate in the United States and in particular amongst people who are in close physical contact, such as football athletes and childcare workers [8]. A recent national estimate for invasive MRSA incidence rates showed one in three people carry *S. aureus* in their nose and two in 100 people carry MRSA. *Enterococci* bacteria have the ability to survive for months in humans and animals. Similar to MRSA, VRE infections are commonly acquired by hospitalized patients. Enterococcal infections can be lethal, particularly those caused by VRE. According to the CDC, the number of nosocomial VRE isolates increased in the United States 20-fold, between 1989 and 1993. VRE is now the second to third most common cause of nosocomial infections in the USA [9].

In order to acquire substantial quantities of machaeriol A-D (**6**–**9**) and machaeridiol A-C (**10**–**12**) for comprehensive antimicrobial evaluations against MRSA and VRE, a reinvestigation was conducted on stem bark, leaves, and roots of the original plant material. During the course of this work, the novel epimeric mixture of bibenzylated furanoid monoterpenes, machaerifurogerol (**1a**), and 5-*epi*-machaerifurogerol (**1b**), together with the known isoflavons (+)-vestitol (**2**) and 7-*O*-methylvestitol (**3**), and pterocarpans (+)-medicarpin (**4**) and 3,8-dihydroxy-9-methoxypterocarpan (**5**), as well as previously isolated [5,6] machaeriol A-C (**6**–**8**) and machaeridiol A-C (**10**–**12**), were isolated. In this study, we report the correction of the previously reported botanical identity of the plant *M. multiflorum*, the structure elucidation of compounds **1**–**5**, and comprehensive antimicrobial activities of compounds **1**–**8** and **10**–**12**.

## 2. Results and Discussion

### 2.1. Botanical Identity of Machaerium sp. (Rimachi 12161)

The regional floras and other relevant publications were consulted for possible botanical identification of the species [7,10]. The voucher specimen (Rimachi 12161) was confirmed to belong to the genus *Machaerium*, based on the morphological features and available field information such as habitat, leaf, inflorescence, and fruit characters. The leaves showed partial similarities with those of *M. leiophyllum* var. *leiophyllum* and *M. glabrum*. However, complete identification of the specimen was not possible due to a lack of information on necessary diagnostic features, such as presence or absence of spines, features of stipules, and floral characters. It is possible that the specimen could represent an un-described taxon. The description (vide infra) is based on a single herbarium specimen and the associated collection information available from the original collection. 

The *Machaerium* sp. (12161) plant was found to grow on sandy soils in open forests in Maynas, near Loreto, Peru, at an altitude of about 140–160 m. It is a woody liana with cylindrical stems, imparipinnate leaves with 17–21 leaflets, green flowers in axillary panicles, and 1-seeded samaroid fruits with a terminal wing showing reticulate venations.

### 2.2. Phytochemical Constituents

**Figure 1:**
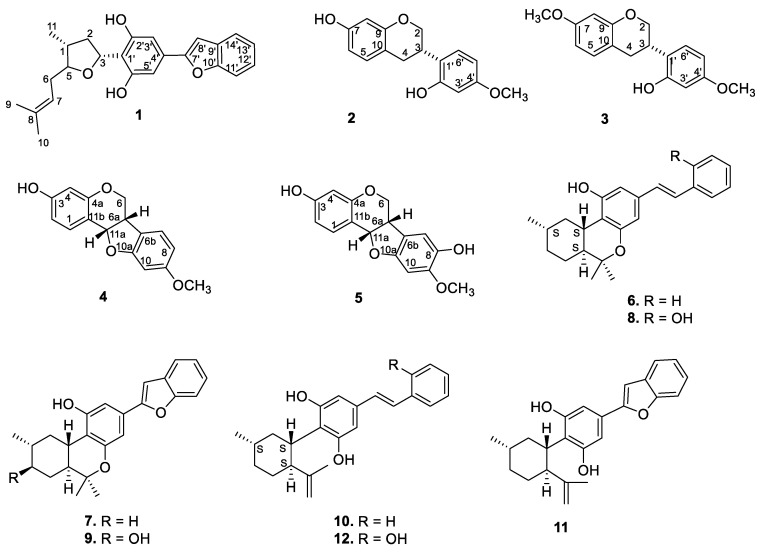
Structures of the isolated compounds.

The dried EtOH extract of the stem bark was fractionated with *n*-hexane, followed by dichloromethane (DCM), and resulted in the isolation of compounds **1**–**5** (Figure 1 and Figure S1) (see the Experimental Section).

**Figure 2:**
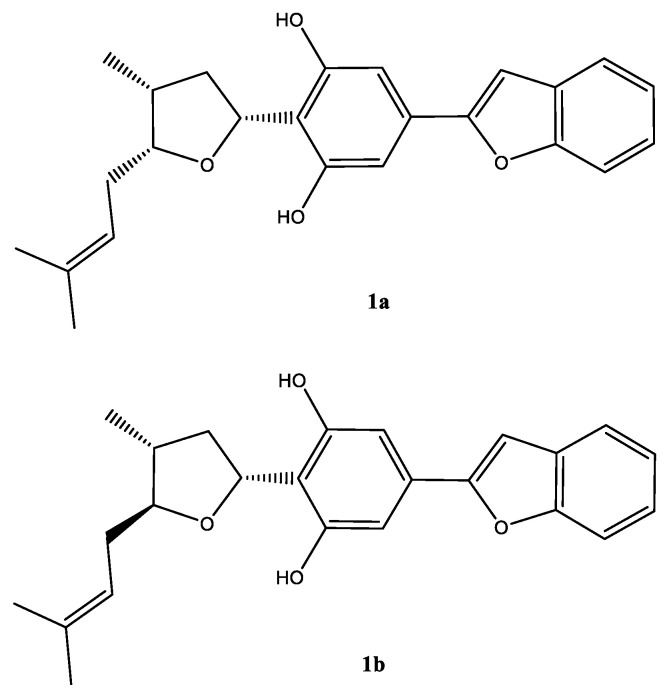
Structure of the epimeric compound (**1a**) (major) and (**1b**) (minor) in mixture.

Chromatographic separation of the DCM fraction 20–24 that led to the isolation of compound **1**, showed a single peak upon LC–MS analysis, which showed a protonated molecular ion peak at m/z 379.1906 [M + H]^+^ in its ESI–HRMS, suggesting the molecular formula C_28_H_29_O_3_. A careful analysis of the ^1^H and ^13^C NMR spectra (Table 1), and 2D NMR COSY, HMQC, HMBC, and NOESY spectra (Figures S2–S11) suggested that the compound was a mixture of C-5 epimers **1a** and **1b** (Figure 2). 

**Figure 3:**
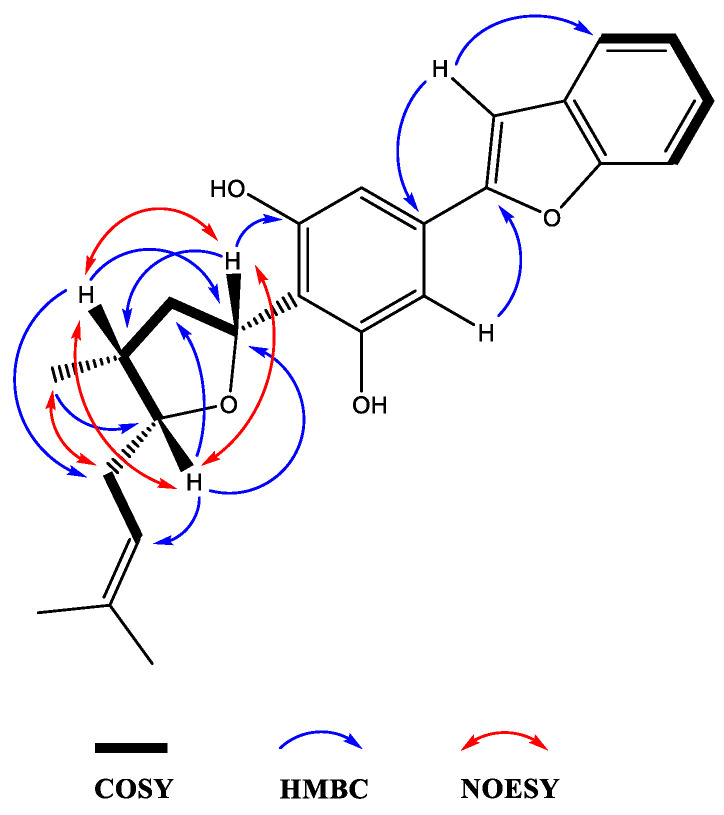
Key 2D NMR COSY, HMBC, and NOESY correlations of compound **1a.**

Moreover, the NMR data were found to be partially comparable with the machaeridiol C (**11**) [6], suggesting the presence of a benzofuran side chain (*δ*_H_ 7.18–7.52) (4H) attached to the substituted resorcinol moiety. The HMBC spectrum (**1a**; Figure 2) showed ^2^*J*- and ^3^*J* correlations between the H-8′ at *δ*_H_ 6.89 (brs) and the two sp^2^-hybridized carbons at C-4′ and C-7′ (*δ*_C_ 130.6 and 154.9, respectively), supporting the attachment of C-4′ of the resorcinol unit to the C-7′ of benzofuran ring. The ^1^H NMR spectrum also showed signals at *δ*_H_ 6.83 (2H) for two identical protons (H-3′ and H-5′), suggesting the presence of C-1′,2′,4′,6′-tetra substituted resorcinol ring with two oxygenated carbons at C-2′ and C-6′ (*δ*_C_ 155.3 and 155.4). In addition, the ^1^H NMR spectrum (**1a**) showed signals at *δ*_H_ 4.0 (1H, m, H-5) and 5.32 (1H, dd, J = 1.2, 7.6 Hz, H-3) for the tetrahydrofuran ring, and at *δ*_H_ 1.02 (3H, d, J = 7.2 Hz, H-11) for a Me-group. The HMBC (Figure 3) spectrum showed correlations between the H-3 (*δ*_H_ 5.32) and the three sp^2^ hybridized carbons at C-1′, C-2′, and C-6′ (*δ*_C_ 113.3, 155.3, and 155.4, respectively), supporting the attachment of the tetrahydrofuran ring at C-3 (*δ*_C_ 77.7) to C-1′ position. The HMBC spectrum also showed cross peaks between the methyl protons H-11 (*δ*_H_ 1.02) and C-1, C-2, and C-5 (*δ*_C_ 35.2, 41.8, and 83.1), supporting the attachment of the methyl (C-11, *δ*_C_ 15.5) to the tetrahydrofuran at C-1 (*δ*_C_ 35.2). The ^1^H, ^13^C, and 2D NMR spectra supported the presence of the 2-methylbut-2-ene unit. This was confirmed by the HMBC spectrum, which showed correlations between the H-6 at (*δ*_H_ 2.4) and the three carbons at C-5, C-7, and C-8 (*δ*_C_ 83.1, 120.0, and 134.5, respectively), confirming the attachment of the methylbut-2-ene unit to the tetrahydrofuran moiety at C-5. The relative configurations at C-1, C-3, and C-5 of the tetrahydrofuran were assigned via careful analysis of NOESY correlations for **1a**. In the NOESY spectrum (assigned for **1a**), H-5 (*δ*_H_ 4.0) showed correlation with H-1 (*δ*_H_ 2.4) and H-3 (*δ*_H_ 5.32), indicating the cofacial (*β*)-orientation of the three groups. Additionally, NOESY showed cross peaks between H-1 (*δ*_H_ 2.4.) and H-3 (*δ*_H_ 5.32), which supported their presence in the same plane of the molecule like H-5. On the other hand, such nOe signals were not evident in the NOESY of the minor compound **1b**. Moreover, in its ^1^H NMR spectrum, H-5 and H-3 were deshielded at δ_H_ 4.21 (1H, m) and 5.59 (1H, dd, J = 12.0, 8.0 Hz), respectively, for the tetrahydrofuran ring, and at *δ*_H_ 1.02 (3H, d, J = 7.2 Hz, H-11) for an Me-group. Moreover, a CD analysis for this compound revealed a weak spectrum in the range of 250–500 nm, which is reflective of the epimeric nature of the compound. Based on the foregoing discussion and comparing the NMR data with compound **11** [6], the structure **1a** and **1b** were determined for machaerifurogerol and 5-*epi*-machaerifurogerol, respectively.

During the course of isolation, four isoflavonoid derivatives (**2**–**5**) and previously reported machaeriols (**6–8**) and machaeridiols (**10**–**12**) were isolated from the DCM partition of the stem bark and leaves extracts. However, the minor compound **9** could not be isolated due to a paucity of material. Compounds **2** and **3** were identified as known isoflavons (+)-vestitol and 7-*O*-methylvestitol, while **4** and **5** were identified as known peterocarpans. (+)-medicarpin and 3,8-dihydroxy-9-methoxy-pterocarpan, respectively, previously reported from *Machaerium vestitum* and Cuban propolis [10,11]. Compounds **4** and **5** were also reported from Cuban propolis [11]. The ^1^H and ^13^C NMR spectroscopic data (see Table S1) of compounds **2**–**5** were in agreement with those reported [11,12]. In addition, examination of the leaves of *Machaerium* sp. also yielded compounds **6**–**8** and **10**–**12**, as well as their presence in the root extract. The identities of compounds **6**–**12** were established by NMR spectra and by direct comparison with authentic samples (TLC, HPLC/ LC–MS).

### 2.3. Antimicrobial Activity against Gram-Positive Species and Fungi

The availability of machaeriols A-C (**6**–**8**) and machaeridiols (**10**–**12**) [5,6] offered the opportunity to carry out a comprehensive investigation of antimicrobial activity. Among the tested fractions, DCM-25-32 (enriched with compounds **8** and **10**–**12**) and DCM-10-15 (enriched with compound **12**) were the most active against bacteria *S. aureus*, and methicillin-resistant *S. aureus* (MRSA), and the fungi *Candida glabrata*, *C. krusei*, and *Cryptococcus neoformans*, with IC_50_ values of <0.8, <0.8, <0.8, 5.35, <0.8 μg/mL, and <0.8, 1.95, 3.0, 6.07, 12.58 μg/mL, respectively (Table 2). Antibacterial activities of **6**–**8, 10**–**12**, and a mixture **10** + **11** (1:1) were evaluated against methicillin-resistant *S. aureus* (ATCC 1708, 1696, and 1717), the ex vivo MRSA XEN31 strain, and vancomycin-resistant *Enterococci* (VRE; *Enterococcus faecium* ATCC 700221), low-level VRE (*E. faecalis* ATCC 51299), and the vancomycin-sensitive strain (VSA; *E. faecalis* ATCC 29212) (Table 3 and Table 4). Compound **11** and mixture **10** + **11** (1:1) showed the most potent activity against MRSA BAA 1696, BAA 1708, BAA 1717, and BBA 33591 with IC_50_/MIC/MBC values of 0.43/1.25/5 μg/mL, 0.38/1.25/1.25 μg/mL, 0.38/1.25/2.5 μg/mL, 0.71/1.25/1.25 μg/mL; and 0.41/1.25/10 μg/mL, 0.34/1.25/1.25 μg/mL, 0.39/1.25/1.25 μg/mL and 0.61/1.25/10 μg/mL, respectively. On the other hand, compound **8** and mixture **10** + **11** were found to be the most potent against *E. faecium* ATCC 700221 and *E. faecalis* ATCC 51299, (VRE) and *E. faecalis* ATCC 29212 (VSE) with IC_50_/MIC/MBC of 0.48/1.25/2.5 μg/mL, 1.02/1.25/5 μg/mL and 1.16/2.5/2.5 μg/mL; and 0.49/1.25/2.5 μg/mL, 0.70/1.25/5 μg/mL, and 0.72/1.25/5 μg/mL, respectively (Table 3 and Table 4). The activities of compounds **6**–**8** and **10**–**12** were found to be more potent than ciprofloxacin and vancomycin against the MRSA BBA 1708 strain, while **6**, **8**, and **10** + **11** were more active against VRE 700221 than the positive controls.

### 2.4. Antimicrobial Combination Studies

In light of the strong antimicrobial activity of the DCM fraction 25–32, which is enriched with compounds **8**, **10**–**12** (Table 3), a combination study using an in vitro Checkerboard method [13,14] was carried out for machaeriol (**7** or **8**) and machaeridiol (**11** or **12**), to evaluate the synergy of combination treatment against the strains of MRSA and *Enterococcus* (VRE) (Table 4). Among these compounds, a combination of machaeridiol B (**12**; at MIC 5 μg/mL) and machaeriol C (**8**; at ½ MIC 1.25 μg/mL) exhibited a potent activity, with the MIC values of 0.156 and 0.625 μg/mL exhibiting a >32- and >8-fold reduction of MICs, compared to those observed for **12**, against MRSA 1708 and MRSA 1717 strains. When these two compounds were tested with an inverse concentration (i.e., MIC of **8**; 2.5 μg/mL + ½ MIC of **12**; 2.5 μg/mL), a strong synergism was also observed, but to a lesser extent. When tested against VRE (*E. faecium* 700221), this combination showed synergism with the MIC values of 1.25 μg/mL, a >4-fold reduction of MIC compared to **12** (Table 5).

**Figure 4:**
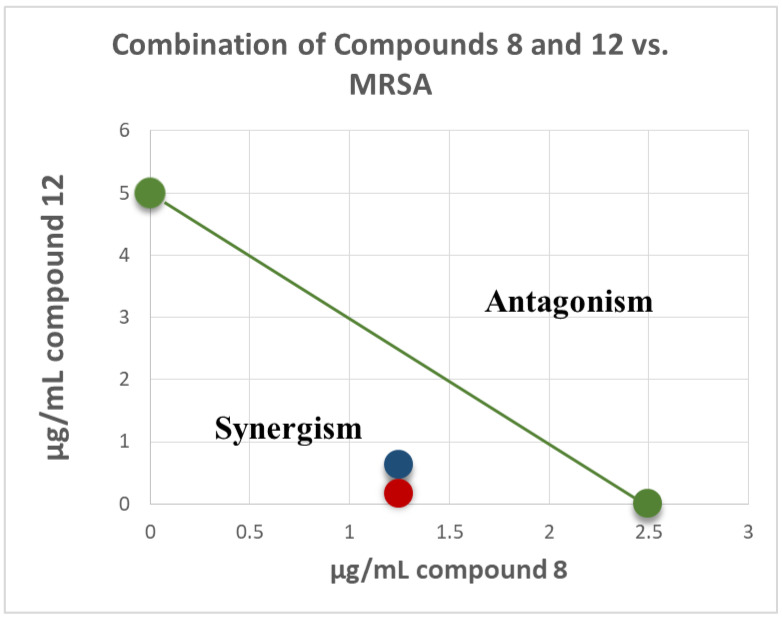
Isobologram of combination of compounds **8** and **12** in MRSA 1708 (red) and 1717 (blue). The green series represents the additivity line of compounds **8** and **12** green dots represent the MIC of each compound alone; the green line represents all possible additive combinations). The red (MRSA 1708) and blue (1717) dots represent the combination of compounds **8** and **12**, and show that they fell below the additivity line (the combination of compounds produces a synergistic effect beyond additivity).

Isobologram showing synergistic activity of the combination of compounds **8** and **12** in MRSA 1708 (red) and 1717 (blue) are presented in Figure 4. The green series represents the additivity line of compounds **8** and **12** (green dots represent the MIC of each compound alone; the green line represents all possible additive combinations). The red (MRSA 1708) and blue (1717) dots represent the combination of compounds **8** and **12**, and show that they fell below the additivity line (the combination of the compounds produces a synergistic effect beyond additivity). This synergism between machaeriol (HHDBP) and machaeridiol (seco-HHDBP) could be due to different molecular targets affected by these two molecules. A combination study of compounds **8** and **12** with antibiotics, either methicillin or ciprofloxacin, did not show any additive or synergistic effects.

### 2.5. Antimicrobial Activity against Gram-Negative Species

The activity of the compounds was determined in the Gram-negative species of the ESKAPEE panel, *Klebsiella pneumoniae, Acinetobacter baumannii, Pseudomonas aeruginosa,* and *Escherichia coli* (Table 6). None of the tested compounds displayed any antimicrobial activity in the Gram-negative species up to a concentration of 128 µg/mL. However, when the outer membrane was permeabilized using the membrane permeabilizer polymyxin-B-nonapeptide (PMBN), MICs as low as 0.5 µg/mL were observed, suggesting an intracellular target. With the exception of **6** + **7**, all compounds tested displayed a good activity in *A. baumannii* and *E. coli* strains and in *P. aeruginosa* strain PAO1. The *P. aeruginosa* strain NCTC 13437 is a near-pan drug resistant strain, and this was reflected in the MICs to compounds **6** + **7**, **10** + **11**, **11**, and **12**, although **8** had an MIC of 8 µg/mL in the presence of PMBN. None of the compounds displayed activity in the two *K. pneumoniae* strains, even in the presence of PMBN, suggesting the target/pathway might be missing or modified in this species.

## 3. Materials and Methods

### 3.1. General Experimental Procedures

Optical rotations were recorded at ambient temperature using a Rudolph Research Analytical Autopol IV automatic polarimeter. IR spectra were obtained using a Bruker Tensor 27 instrument. NMR spectra were acquired on a Varian Mercury 400 MHz NMR spectrometer at 400 (^1^H) and 100 MHz (^13^C) in CDCl_3_, using the residual solvent as an internal standard. Multiplicity determinations (DEPT) and 2D NMR spectra (HMQC, HMBC, NOESY) were obtained using the standard Bruker pulse programs. ESI-HRMS were acquired by direct injection using a Water Xevo G2-S TOF with electrospray ionization (ESI). TLC was carried out on pre-coated silica gel 60 F254 (EMD Chemicals Inc, Darmstadt, Germany) using toluene-EtOAc (9:1) and *n*-hexane-EtOAc (7.5:2.5) as solvents. Centrifugal preparative TLC (CPTLC, using a Chromatotron, Harrison Research Inc. model 8924, tagged with a fraction collector) was carried out on 6 mm custom-made RP C_18_ silica gel [15], and silica gel P_254_ (Analtech) 1, 2, and 4 mm rotors, using H_2_O-MeOH, EtOAc-:*n*-hexane, and CHCl_3_ as eluents. SPE cartridges C_18_ (Supelco Inc., Bellefonte, PA, USA) were used in the fractionation work. Purifications were performed on prep-HPLC (silica gel-100 A 250 × 15.00, 5 µM; Phenomenex Luna, Torrance, CA, USA) using an HPLC Delta Prep 4000 equipped with a dual wavelength detector Model 2487 adjusted at 210 and 254 nm (Waters Corporation, Milford, MA, USA), Preparative HPLC was carried out on Waters LC module I plus, using Phenomenex C18, 22 mm, λ 254, flow 15 mL/min, 0–2 min [90% H_2_O; 10% MeCN], 2–45 min, 10% MeCN→ 100% MeN, 45–50 min 100% MeCN]. Samples were dried using a Savant Speed Vac Plus SC210A concentrator. The compounds were visualized by spraying the TLC plates with 1% vanillin-H_2_SO_4_ spray reagent. 

### 3.2. Plant Material

The stem bark, leaves, and roots of *Machaerium* Pers. (Manuel Rimachi, Y.-12161), previously identified as *M*. *multiflorum* Spruce [5] by (Late) Professor Sydney T. McDaniel, was collected in November, 1997, from open sandy forest near Loreto (Maynas), Peru. The voucher specimen (Manuel Rimachi Y. 12161) is deposited at the Missouri Botanical Garden (http://www.tropicos.org/Specimen/100326687).

### 3.3. Extraction and Isolation of Compounds from Stem Bark and Leaves

The powdered stem bark (0.5 kg) was extracted by percolation with 95% EtOH (3 × 2 L) and the combined extracts were evaporated under reduced pressure (yield 17.7 g). A portion of the dried EtOH extract (15 g) was percolated with *n*-hexane, followed by DCM, and finally the residual extract was washed with MeOH (each 200 mL × 3). The *n*-hexane, DCM, and MeOH fractions were separately filtered and dried, which afforded 3.8, 8.9, and 4.5 g, respectively. The antimicrobial activity was detected in the DCM fraction (IC_50_ < 20 *μ*g/mL against *S. aureus* and MRSA). A portion of the dried DCM fraction (1.65 g) was fractionated by CPTLC with a Chromatotron® instrument, using a 4 mm custom-made C_18_ RP silica gel ChromatoRotor^TM^ [15], eluting with a gradient of 60% to 100% MeCN-H_2_O to afford 30 fractions. The fractions were pooled by TLC analyses.

Fractions 1–8 (475 mg) were combined and further subjected to CPTLC, using a 4 mm silica gel P_254_ disc, and gradient elution with MeCN:DCM. Elution with 2% MeCN:DCM afforded medicarpin (**4**; 4.5 mg), followed by elution with 4% MeCN:DCM, which gave 3,8-dihydroxy-9-methoxy-pterocarpan (**5**; 7.5 mg), and finally elution with 5% MeCN:DCM yielded vestitol (**2**; 9.8 mg). The combined fractions 10–15 (70 mg) was subjected to preparative C_18_ RP-HPLC, using 90% MeCN:H_2_O as solvent, which afforded machaeridiol B (**12**), followed by machaeridiol A (**10**) and machaeridiol C (**11**). Similarly, combined fractions 25–32 (100 mg) was also separated by preparative C_18_ RP-HPLC, which afforded additional quantities of **10**–**12** [total yields: **10** (10 mg), **11**; (18 mg), **12** (21 mg)] and machaeriol C (**8**; 34.6), however, the minor compound machaeriol D (**9**) could not be re-isolated due to a paucity of material. Further elution with 75% MeCN:H_2_O afforded 13 fractions, which contained the mixture of two compounds **6** + **7**, (50 mg). The mixture was then separated by preparative C_18_ RP-HPLC (column: ODS prodigy 10µ, 250 × 10 mm; detector: UV-254 nm), using 95% MeCN:H_2_O as solvent, which afforded **6** (16 mg), followed by **7** (16 mg). Finally, the dried *n*-hexane fraction (77 mg) was subjected to CPTLC, using a 2 mm C_18_ RP rotor, and eluted with 65% MeCN:H_2_O, which afforded 7-*O*-methylvestitol (**3**; 8 mg). A sub-fraction of DCM (15 mg) was subjected to prep-HPLC (Waters LC module I plus, using Phenomenex C_18_, 2 mm), which afforded compound **1a**+**1b** (5 mg). The structures of (+)-vestitol (**2**), 7-*O*-methylvestitol (**3**), (+)-medicarpin (**4**) and 3,8-dihydroxy-9-methoxypterocarpan (**5**) were determined by physical and spectroscopic data (^1^H and ^13^C NMR, see SI 1), and also by comparison with those reported [11,12]. The structures of the re-isolated compounds **6**–**8** and **10**–**12** were identified by NMR data [5,6] and by direct comparison (TLC, HPLC/LC–MS) with their respective authentic samples available in our laboratories. Finally, powdered leaves (560 g) and root bark (50 g) of *Machaerium* sp. were extracted using the method described previously [5,6], and compounds 6–8 and 10–12 were isolated from leaves as describe below. 

The powdered leaf was percolated with *n*-hexane, followed by DCM and EtOH (each 3 × 2 L) to yield 5, 14, and 9 g of extracts, respectively. A portion of the DCM extract (10 g) was subjected to reversed phase (RP) cartridge (10 G, 60 mL Giga tube), and eluted with MeCN-H_2_O to afford 30 fractions. The combined fractions 20–21 (102 mg; eluted by 60–65% MeCN-H2O) were subjected to centrifugal preparative thin layer chromatograph (CPTLC, 1 mm Si gel P254 disc), eluting with 0.5–1% MeCN-DCM to yield **12** (11.4 mg). Fraction 22 (60 mg; eluted with 75% MeCN-H_2_O) was further subjected to CPTLC (1 mm RP-C18 ChromatoRotor), eluted with 50–100% H_2_O-CH_3_CN to afford 115 fractions, of which fractions 42–45 and 90–115 eluting with 80% and 90% MeCN-H_2_O yielded **10** (2.7 mg) and **11** (4.5 mg), respectively. Combined fractions 46–89 (28.4 mg) were enriched with **12** (+ traces of **10** + **11**). Similarly, RP cartridge purified fractions 23 and 24 (60 and 70 mg; eluted with 70 and 80% MeCN-H_2_O, respectively) were further purified (1 mm RP-C18 ChromatoRotor) by eluting separately with 50–100% H_2_O-MeCN to yield a mixture of **8** + **10** + **11** (32 mg and 31 mg respectively). The above enriched mixtures were further purified preparative RP-HPLC, using 90% MeCN-H_2_O as solvent to afford compounds **12**, **11** + **12**, **11**, and **8** (5, 32, 4 and 31.7 mg, respectively). 

A portion of *n*-hexane extract (2.5 g) was fractionated with CPTLC (6 mm, Si gel P254 disc) eluting with 5% DCM in hexane to yield 10 fractions. The fractions 3–7 (840 mg) that enriched with compounds **6** and **7** were combined and further attempted to purify with an additional CPTLC (4 mm, Si gel P254 disc) eluting with 5% DCM in hexane to yield semi-pure **6** (40 mg), **6**+**7** (50 mg), and semi-pure **7** (6 mg). In addition, the presence of these compounds in leaves, stem bark, and root extracts were confirmed by HPLC and LC–MS (vide infra).

### 3.4. Machaerifurogerol (***1a***) and 5-epi-Machaerifurogerol (***1b***)

Amorphous solid; [*α*]^26^_D_ +5.8 (*c* 0.05, MeOH); IR (KBr) *υ* max 3341 (OH), 2924, 1631, 1574, 1452, 1248, 961, 801, 613, 591 cm^−1^; ^1^H and ^13^C NMR, see Table 1; HRESIMS m/z 379.1906 [M + H]^+^ (calcd. for C_24_H_27_O_4_, 379.1865).

### 3.5. Identification of Compounds **6**–**8** and **10**–**12** by LC–MS

LC–MS analysis was carried out on an Agilent system using Luna 5 μ C18 (2), 150 × 4.6 mm, λ 254, flow 1 mL/min, gradient 0–2 min [95% H_2_O; 5% MeCN], 2–30 min, 5% MeCN→ 100% MeCN, 30–35 min 100% MeCN, 35–45 min [95% H_2_O; 5% MeCN]. The retention times (*R_t_*) of the compounds **6** (m/z 349.2 [M + H]^+^; C_24_H_29_O_2_), **8** (365.2 [M + H]^+^; C_24_H_29_O_3_), **7** (363.2 [M + H]^+^; C_24_H_27_O_3_), **10** (349.2 [M + H]^+^; C_24_H_29_O_2_); **11** (363.2 [M + H]^+^; C_24_H_27_O_3_), and **12** (365.2 [M + H]^+^; C_24_H_29_O_2_) were found to be 4.4, 4.5, 9.9, 10.0, 9.4, and 9.7 min^−1^, respectively. Compounds **6**–**9** and **10**–**12** were identified from leaves, stem bark, and root extracts through HPLC and LC–MS.

### 3.6. Antimicrobial Assays

All organisms were obtained from the American Type Culture Collection (Manassas, VA, USA) or the National Collection of Type Cultures (Colindale, UK), unless specified otherwise. These included the yeasts *Candida albicans* ATCC 90028, *C. glabrata* ATCC 90030, and *C. krusei* ATCC 6258; the fungi *Cryptococcus neoformans* ATCC 90113 and *Aspergillus fumigatus* ATCC 204305; and the bacteria *Escherichia coli* ATCC 35218, NCTC 12923, *Klebsiella pneumoniae* NCTC 13368, M6 (Colindale, UK), *Acinetobacter baumannii* AYE (ATCC BAA-1710), ATCC 17978, *Pseudomonas aeruginosa* ATCC 27853, PAO1 (Manoil collection, University of Washington, Washington, DC, USA), NCTC 13437, *Mycobacterium intracellulare* ATCC 23068, methicillin-resistant *Staphylococcus aureus* ATCC 33591 (MRSa), USA-300 MRSa (ATCC BAA-1717), USA-400 MRSa (ATCC BAA-1696), Mupirocin-resistant *S. aureus* (ATCC BAA-1708), *Enterococcus faecium* ATCC 700221 (VRE), *E. faecalis* ATCC 29212 (Vancomycin-sensitive) and *Enterococcus faecium* ATCC 51299 (Vancomycin-intermediate). Drug controls ciprofloxacin, methicillin and vancomycin (ICN Biomedicals, Aurora, OH, USA) for bacteria and amphotericin B (ICN Biomedicals) for yeasts and fungi were included in each assay. Susceptibility testing was performed using a modified version of the CLSI (formerly NCCLS) method [16,17,18]. *M. intracellulare* was tested using a modified Franzblau method [18]. Samples were serially diluted in 20% DMSO/saline and transferred in duplicates to 96-well flat-bottomed microplates. Microbial inocula were prepared by correcting the OD_630_ of microbe suspensions in incubation broth to give final target inocula. All organisms were read at either 530 nm, using the Biotek Powerwave XS plate reader (Bio-Tek Instruments, Winooski, VT, USA) or 544ex/590em, (*M. intracellulare, A. fumigatus*) using the Polarstar Galaxy Plate Reader (BMG Lab Technologies, Ortenburg, Germany), prior to and after incubation. Minimum fungicidal or bactericidal concentrations were determined by removing 5 μL from each clear well, followed by transferring to agar, and incubating. The MFC/MBC was defined as the lowest test concentration that kills the organism (allows no growth on agar).

Gram-negative MICs were determined using the CLSI microbroth dilution method, modified as described previously [19]. Bacteria were added at a starting concentration of 5 × 10^5^ cfu/mL and incubated for 20 h at 37 °C in the dark. Absorbance at OD_600_ was then read using the CLARIOstar plate reader (BMG Lab Technologies, Germany). The MIC was defined as the lowest concentration where visible growth could not be detected, equivalent to an OD_600_ of 0.1. MICs were also determined in the presence of the membrane permeabilizer, polymyxin-B-nonapeptide (PMBN) following the same method, with an additional step; after the 2-fold dilution of compound was prepared and before the bacteria were added, PMBN was added to all wells at a final concentration of 30 µg/mL. This concentration was shown to not significantly inhibit growth of the test panel.

### 3.7. Antimicrobial Combination Study by Checkerboard Method

The combination study of the compounds was carried out using a standard Checkerboard method [13,14]. Strains were grown on Eugon agar at 35 °C, prior to assays. Test samples were dissolved in DMSO (2 mg/mL) to the desired concentrations, and serially-diluted with 20% DMSO/saline. Samples were transferred to 96 well assay plates (10 µL) in a checkerboard layout. Inocula were prepared by suspending growth from agar in 0.9% saline, determining the OD_630_, and correcting in incubation broth (cation-adjusted Mueller-Hinton, Difco) to afford 5 × 10^5^ colony forming units per mL, after addition to samples (180 µL) using standard inocula calculations. Final sample test concentrations were 1/100th the DMSO stock concentrations. The assay plates were read at 530 nm prior to and after incubation at 35 °C for 18–20 h. IC_50_s of each test compound were calculated using the XLfit 4.2 software (IDBS, Alameda, CA, USA) using the fit model 201. After incubation, all 96 wells were also pinned to Eugon Agar and incubated at 35 °C overnight to determine bactericidal activity. Fractional inhibitory concentrations (FICs) were calculated to evaluate possible synergy with FICS < 0.5 synergistic.

## 4. Conclusions

Based on our investigation carried out on the identity of the plant, and a re-examination of the voucher specimen (Rimachi # 12161) at the MOBOT, it can now be concluded that this species should be treated only as an unidentified species of *Machaerium* Pers., as determined by the collection information (Manuel Rimachi, Y. 12161). This appears to be the first report of macharifurogerol (**1a**) and its epimer **1b** from a natural source. In addition, the isolation of isoflavons (**2** and **3**) and pterocarpans (**4** and **5**) from this *Machaerium* species (Rimachi 12161) illustrated that these isoflavonoids are typical chemotaxonomic markers of the genus *Machaerium* [11,12]. Machaeriols and its biogenetic precursor machaeridiols are only isolated from this species (#12161) from the genus *Machaerium*, which are analogous to hexahydrocannabinol (HHC) and dihydrocannabidiol base skeletons of *Cannabis* and its variants, in higher plants [6]. The only other bibenzyl analogue of Δ^9^-THC, perrottetinen, was previously reported from the liverwort *Radula perrottetii* [20]. It is intriguing to note that the strong MRSA and VRE inhibitory activities, together with their antiparasitic activities [5,6] of the isolated compounds of *Machaerium* (12161) is contributed by HHDBP machaeriols and their 5,6-*seco* analogs machaeridiols. The stereo-specific total synthesis of machaeriol A-D (**6**–**9**) and mechaeridiol B (**12**) was reported [21,22,23,24]. In addition, analogs of machaeriols and related HHC were recently synthesized, which showed anticancer activity [25]. It was anticipated that these phytocannabinoids could serve as potential template for anti-MRSA and anti-VRE lead candidates, because of their inherent inhibitory activities alone, as well as strong synergistic activity when tested in combination with machaeriol and machaeridiol. The observation of significant activity in permeabilized multidrug resistant Gram-negative pathogens, also offers the potential for optimization of the chemical scaffold to generate analogues with better cell permeability. These compounds might provide important new leads for WHO priority Gram-negative bacterial pathogens.

## Figures and Tables

**Table 1:** ^1^H and ^13^C NMR (in CDCl_3_) data for epimeric compounds **1a** and **1b**.

Position	1a (Major Epimer)		1b (Minor Epimer)		HMBC
	*δ*c ^a^ (*J* in Hz)	*δ*_H_^b^ (*J* in Hz)	*δ*c ^a^ (*J* in Hz)	*δ*_H_^b^ (*J* in Hz)	
1	35.2 CH	2.4 (m)	35.4 CH	2.4 (m)	5, 2, 11
2	41.8 CH_2_	2.72 (m), 1.59 (m)	42.0 CH_2_	2.39 (m), 1.58 (m)	5, 11
3	77.7 CH	5.32 (dd, 12, 7.6)	77.4 CH	5.59 (dd, 12, 8.0)	2, 1’, 2’, 6’
5	83.1 CH	4.00 (m)	83.3 CH	4.21 (m)	11, 7
6	29.9 CH_2_	1.24 (br s), 2.4 (m)	29.4 CH_2_	1.24 (br s), 2.4 (m)	7, 8, 5
7	120.0 CH	5.18 (t, 7.2)	120.0 CH	5.18 (t, 7.2)	9, 10
8	134.5 C		134.4 CH		
9	18.2 CH_3_	1.66 (s)	18.3 CH_3_	1.66 (s)	7, 8, 10
10	26.1 CH_3_	1.72 (s)	26.1 CH_3_	1.72 (s)	7, 8, 9
11	15.5 CH_3_	1.02 (d, 7.2)	13.9 CH_3_	1.03 (d, 7.2)	1, 2, 5
1’	113.3 C		114.0 C		
2’	155.3 C		155.3 C		
3’	104.9 CH	6.83 (br s)	104.9 CH	6.83 (br s)	5’, 4’, 1’
4’	130.6 C		130.8 C		
5’	104.9 CH	6.83 (br s)	104.9 CH	6.83 (br s)	3’, 4’, 1’, 7’
6’	155.4 C		155.4 C		
7’	154.9 C		154.9 C		
8’	101.7 CH	6.89 (br s)	101.6 CH	6.89 (br s)	4’, 7’
9’	129.5 C		129.4 C		
10’	155.5 C		155.5 C		
11’	111.3 CH	7.45 (d, 7.6)	111.3 CH	7.45 (d, 7.6)	12’, 13’, 10’
12’	124.4 CH	7.22 (m)	124.4 CH	7.22 (m)	13’, 11’, 10’
13’	123.1 CH	7.18 (m)	123.1 CH	7.18 (m)	11’, 9’
14’	121.1 CH	7.52 (d, 7.2)	121.1 CH	7.52 (d, 7.2)	8’, 10’, 13’

^a 1^H Spectra recorded at 400 MHz, and ^b 13^C spectra recorded at 100 MHz.

**Table 2:** Antimicrobial activity (IC_50_ in μg/mL) of *Machaerium* DCM fractions ^a^.

	*C. glabrata*	*C. krusei*	*C. neoformans*	*S. aureus*	*MRSA*	*VRE* 29212 ^b^	*VRE* 51299 ^c^	*VRE* 700221 ^d^
DCM-1-8	>20	>20	>20	6.09	5.64	-	-	-
DCM-10-15	>20	>20	4.26	<0.8	0.81	11.41	1.89	3.47
DCM-12	6.07	12.58	<0.8	1.95	3	5.68	1.65	2.95
DCM-25-32	<0.8	5.35	<0.8	<0.8	<0.8	3.70	1.34	<0.8
DCM-56	>20	>20	>20	>20	>20	-	-	2.94
DCM-1-8-A-31	>20	>20	>20	9.91	>20	-	-	-
DCM-1-8-A-41	>20	>20	4.41	4.51	10.62	5.41	8.52	4.52
DCM-1-8-A-63	>20	>20	>20	4.29	4.55	11.39	14.49	9.97
DCM-1-8-A-167	>20	>20	>20	4.5	4.41	-	-	-

^a^ IC_50_ is the concentration causing 50% growth inhibition; MIC (minimum inhibitory concentration) is the lowest concentration that allows no growth; MFC (minimum fungicidal concentration) or MBC (minimum bactericidal concentration) is the lowest concentration at kills the test organism; ^b^ Vancomycin sensitive, ^c^ Low-level vancomycin resistant. ^d^ Vancomycin resistant strain; -: not active at the highest test concentration of 20 µg/mL.

**Table 3:** Anti-MRSA activities (in μg/mL) of compounds **6**–**8** and **10**–**12**.

	MRSA BAA-1708	MRSA BAA-1717	MRSA 33591	MRSA BAA-1696	MRSA XEN31
Compound	IC_50_/MIC/MBC	IC_50_/MIC/MBC	IC_50_/MIC/MBC	IC_50_/MIC/MBC	IC50/MIC/MBC
**6**	10.61/-/-	I1.93/-/-	_	11.87/-/-	3.50/-/-
**7**	5.36/-/-	3.29/-/-	NT	14.64/-/-	4.95/20/20
**8**	0.69/1.25/1.25	0.72/1.25/10	NT	0.71/2.5/2.5	0.46/1.25/2.5
**10**	1.03/2.5/5	1.03/2.5/5	NT	1.07/2.5/2.5	1.01/2.5/5
**11**	0.38/1.25/1.25	0.38/1.25/2.5	0.71/1.25/1.25	0.43/1.25/5	0.33/0.63/1.25
**12**	1.52/5/5	0.38/2.5/5	1.57/2.5/5	1.40/5/10	1.64/5/10
**10** + **11**	0.34/1.25/1.25	0.39/1.25/1.25	0.61/1.25/10	0.41/1.25/10	0.32/0.63/1.25
Ciprofloxacin	-/-/-	0.14/0.63/1.25	0.04/0.16/0.31	6.17/-/-	0.10/0.31/0.63
Vancomycin	>20/>20/>20	0.73/1.25/>20	0.47/1.25/5.0	0.37/0.62/>20	NT
Methicillin	2.2/50/50	-	-	2.54/50/50	0.38/1.56/3.13
Cefotaxime	0.35/0.63/0.63	0.35/0.63/0.63	NT	2.47/12.5/25	0.29/0.78/3.13

NT not tested.

**Table 4:** Anti-VRE activities (in μg/mL) of compounds **6**–**8** and **10**–**12**.

	*Enterococcus faecalis* ATCC 29212 ^a^	*Enterococcus faecalis* ATCC 51299 ^b^	*Enterococcus faecium* ATCC 700221 ^c^
Compound	IC_50_/MIC/MBC	IC_50_/MIC/MBC	IC_50_/MIC/MBC
**6**	3.51/-/-	18.79/-/-	0.55/1.25/10
**8**	1.16/2.5/2.5	1.02/1.25/5	0.48/1.25/2.5
**12**	2.99/5/10	2.96/5/10	1.91/2.5/5
**10** + **11**	0.72/1.25/5	0.70/1.25/5	0.49/1.25/2.5
Ciprofloxacin	0.25/0.78/6.25	0.22/0.39/6.25	>20/>20/>20
Vancomycin	0.73/1.25/>20	3.8/10/>20	>20/>20/>20
Methicillin	15.3/25/50	14.2/50.0/50.0	>20/>20/>20

^a^ vancomycin sensitive; ^b^ low-level vancomycin resistant; ^c^ vancomycin resistant.

**Table 5:** Combination study (MIC in μg/mL) ^a^ of compounds **7**, **8, 11**, and **12** by Checkerboard assay against MRSA and VRE.

Compound	MRSA 1708	MRSA 1717	MRSA 33591	MRSA 1696	MRS XEN31	Ef 29212 ^b^	Ef 51299 ^c^	Ef 700221 ^d^
**7**	20	>10	NT	10	20	NT	NT	NT
**8**	2.5	2.5	2.5	2.5	1.25	2.5	2.5	1.25
**12**	5	5	5	5	5	5	>5	5
**11**	20	20	NT	10	10	NT	NT	NT
**8** + **12** (+2.5 µg/mL)	0.625 (↓4X)	0.625 (↓4X)	1.25 (↓2X)	0.625 (↓4X)	NT	1.25 (↓2X)	1.25 (↓2X)	0.625 (↓2X)
**12** + **8** (+1.25 µg/mL)	0.156 (↓32X)	0.625 (↓8X)	1.25 (↓4X)	NT	NT	2.5 (↓2X)	2.5 (↓2X)	NT
**12** + **8** (+0.625 µg/mL)	NT	NT	NT	2.5 (↓2X)	NT	NT	NT	1.25 (↓4X)
**11** + **7** (+2.5 µg/mL)	5.0 (↓4X)	10.0 (↓2X)	NT	5.0 (↓2X)	2.5 (↓4X)	NT	NT	NT
DAPG *^e^*	>1.25	NT	NT	NT	NT	NT	NT	>1.25
**8** + DAPG (+0.63 µg/mL)	2.5 (=)	NT	NT	NT	NT	NT	NT	1.25 (=)
**12** + DAPG (+0.63 µg/mL)	>5 (=)	NT	NT	NT	NT	NT	NT	5 (=)
Methicillin	50	-	-	50	1.56	25	50.0	>20
Vancomycin	>20	1.25	1.25	0.62	NT	1.25	10	>20

^a^ In general, when the MIC of each compound decreased ≥4X in the presence of the other, it is considered synergistic; reduction of MIC in parentheses; ^b^ Vancomycin-sensitive *Enterococcus faecalis*; ^c^ Low-level vancomycin-resistant *Enterococcus faecium*; ^d^ Vancomycin-resistant *Enterococcus faecium*; *^e^* Diacetylphloroglucinol; NT: Not tested.

**Table 6:** Gram-negative MIC Values (in µg/mL) of compounds **6**–**8** and **10**–**12** in the presence of the membrane permeabilizer polymyxin-B-nonapeptide at 30 µg/mL.

	*Klebsiella pneumoniae* NCTC13368	*Klebsiella pneumoniae* M6	*Acinetobacter baumannii* AYE	*Acinetobacter baumannii* ATCC17978	*Pseudomonas aeruginosa* PAO1	*Pseudomonas aeruginosa* NCTC13437	*Escherichia coli* NCTC12923
Compound	MIC	MIC	MIC	MIC	MIC	MIC	MIC
**6** + **7** +PMBN	>64	>64	>64	>64	32	64	>64
**10** + **11** +PMBN	>64	>64	8	0.5	2	64	2
**11** + PMBN	>64	>64	2	1	4	64	2
**12** + PMBN	>64	>64	4	2	16	64	2
**8** + PMBN	>64	>64	8	2	2	8	2
Ciprofloxacin ^a^	0.5	≤0.125	128	0.5	0.5	64	≤0.125
Ceftazidime ^a^	>128	0.25	>128	<0.5	1	>128	0.5
Gentamicin ^a^	4	0.25	>512	0.5	4	256	1

^a^ MICs in the absence of PMBN.

## Supplementary Materials

The following are available online, NMR and HRMS spectra (Figures S1–S11) of compound **1**, and Table for NMR data (Table S1) of compounds **2**–**5** are provided in supporting information.
