# Supplementary material for: Antimicrobial Constituents from Machaerium Pers.: Inhibitory Activities and Synergism of Machaeriols and Machaeridiols against Methicillin-Resistant Staphylococcus aureus, Vancomycin-Resistant Enterococcus faecium, and Permeabilized Gram-Negative Pathogens
Source: Molecules. 2020 Dec 18;25(24):6000. doi: 10.3390/molecules25246000 (PMC7765828; doi:10.3390/molecules25246000)
Supplement: Supplementary file 1 [file molecules-25-06000-s001.pdf]

SUPPORTING INFORMATION

Antimicrobial constituents from *Machaerium* Pers.: Inhibitory activities and synergism of machaeriols and machaeridiols against methicillin-resistant *Staphylococcus aureus*, Vancomycin-resistant *Enterococcus faecium* and permeabilized Gram-negative pathogens

Ilias Muhammad[1,*], Melissa R. Jacob[1], Mohamed A. Ibrahim[1], Vijayasankar Raman[1], Mallika Kumarihamy[1], Mei Wang[1,2], Taha Al-Adhami[3], Charlotte Hind[4], Melanie Clifford[4], Bethany Martin[4], Jianping Zhao[1], J. Mark Sutton[4], Khondaker Miraz Rahman[3,*]

[1] National Center for Natural Products Research, Research Institute of Pharmaceutical Sciences, School of Pharmacy, University of Mississippi, University, MS 38677, United States; mjacob@olemiss.edu (M.R.J; mmibrahi@olemiss.edu (M.A.I); vraman@olemiss.edu (V.R); mkumarih@olemiss.edu (M.K);

[2] Natural Products Utilization Research Unit, Agricultural Research Service, U.S. Department of Agriculture, University, Mississippi 38677, USA; meiwang@olemiss.edu (M.W)

[3] Institute of Pharmaceutical Sciences, School of Cancer and Pharmaceutical Sciences, King's College London, Franklin-Wilkins Building, 150 Stamford Street, London SE1 9NH, UK; taha.al-adhami@kcl.ac.uk (T.A-A);

[4] National Infection Service, Public Health England, Manor Farm Road, Salisbury, SP4 0JG, UK; Charlotte.Hind@phe.gov.uk (C.H); Melanie.Clifford@phe.gov.uk (M.C); Bethany.martin@phe.gov.uk (B.M)

* Correspondence: milias@olemiss.edu; Tel.: +01-662-915-1051 (I.M.); Miraz.rahman@kcl.ac.uk; Tel.: +44 20- 7848 1891 (KMR)

Supporting Information Table

SI #	Title
Table S 1	^{1}H and ^{13}C NMR data for compound **2-5**, (δ, ppm in CD_3OD)
Figure S 1	Structures of isolated compounds
Figure S 2	^{1}H NMR spectrum of compound **1**
Figure S 3	^{1}H NMR spectrum (expansion) of compound **1**
Figure S 4	^{13}C NMR spectrum of compound **1**
Figure S 5	^{13}C NMR DEPT-135 spectrum of compound **1**
Figure S 6	2D NMR HMQC spectrum of compound **1**
Figure S 7	2D NMR HMBC spectrum of compound **1**
Figure S 8	2D NMR COSY spectrum of compound **1**
Figure S 9	2D NMR NOESY spectrum of compound **1**
Figure S 10	EIC (*m/z* = 379) chromatogram of compound **1**
Figure S 11	HRMS spectrum of peak at 6.71 min of compound **1**

Table S 1. ^{1}H and ^{13}C NMR data for compound **2-5** and their lit. data* (δ, ppm in CD_3OD)

	2				3				4				5			
no.	^{1}H	Lit.* data	^{13}C	Lit.* data	^{1}H	Lit.* data	^{13}C	Lit.* data	^{1}H	Lit.* data	^{13}C	Lit.* data	^{1}H	Lit.* data	^{13}C	Lit.* data
1	-	-	-		-		-		7.34(1H, d, 8 Hz)	7.31	132.4	133.2	7.20 (1H, d, 8 Hz)	7.30	133.3	133.0
2	4.01, 3.80 (1H each,m)	4.17, 3.83	71.3	70.9	3.73, 4.20 (1H each,m)	4.22, 3.85	71.3	71.2	6.53 (1H, d, 8 Hz)	6.52	110.1	110.7	6.45 (1H, d, 8 Hz)	6.53	110.8	110.4
3	3.30(1H, m)	3.40	32.3	32.7	3.27 (1H, m)	3.41	33.2	33.1	-		157.5	160.1	-		160.1	159.8
4	2.80, 2.64 (1H each,m)	2.81, 2.66	31.5	31.0	2.85, 2.75 (1H each,m)	2.86 2.70	31.4	31.3	6.40(1H, d, 2 Hz)	6.33	103.9	104.1	6.27(1H, s)	6.33	104.2	103.9
4a	-		-		-		-		-		156.8	158	-		158.1	157.8
5	6.73 (1H, d, 8 Hz)	6.79	131.3	131.6	6.88 (1H, d, 8 Hz)	6.90	131.3	131.2	-		-		-		-	
6	6.22 (1H, m)	6.32	109.1	109.2	6.32 (1H, m)	6.46	108.0	107.9	4.20, 3.60 (1H each,m)	4.23, 3.56	66.6	67.6	4.12, 3.45 (1H each,m)	4.24, 3.59	67.7	67.3
6a	-		-		-		-		3.60 (1H, m)	3.57	40.4	40.9	3.45 (1H, m)	3.57	41.8	41.4
6b	-		-		-		-		-		119.3	120.9	-		119.6	119.2
7	-		157.9	156.9	-		160.9	160.8	7.11(1H, d, 8Hz)	7.18	125.0	126.0	6.70 (1H, s)	6.81	112.6	112.2
8	6.07 (1H, d, 2 Hz)	6.25	103.9	103.6	6.32 (1H, d, 2.0 Hz)	6.38	102.6	102.5	6.43(1H, d, 8 Hz)	6.48	106.6	107.3	-		141.7	141.5

9	-		157.0	156	-		157.4	157.3	-		161.3	162.6	-		149.6	149.1
10	-		115.4	115.4	-		116.1	116.0	6.43(1H, d, 2 Hz)	6.40	97.1	97.6	6.45 (1H, s)	6.51	96.4	96.0
10a	-		-		-		-		-		160.8	162.0	-		154.2	153.8
11	-		-		-		-		-		-		-		-	
11a	-		-		-		-		5.46 (1H, m)	5.48	78.7	80.1	5.38 (1H, m)	5.43	79.7	79.3
11b	-		-		-		-		-		112.6	112.9	-		113.2	112.8
1’	-		121.2	121.5	-		121.4	121.3	-		-		-		-	
2’	-		157.7	156.7	-		156.6	156.5	-		-		-		-	
3’	6.24 (1H, m)	6.38	102.5	101.7	6.32 (1H, m)	6.40	102.5	102.3	-		-		-		-	
4’	-		158.4	160.4	-		160.6	160.5	-		-		-		-	
5’	6.16 (1H, 8 Hz)	6.28	105.8	105.7	6.25 (1H, m)	6.27	105.9	105.8	-		-		-		-	
6’	6.83 (1H, 8 Hz)	6.84	128.9	128.7	6.87(1H, 8 Hz)	6.85	128.9	128.8	-		-		-		-	
MeO-7	-		-		3.61(3H, s)	3.67	55.7	55.6	-		-		-		-	
MeO-9	-		-		-		-		3.80 (3H, s)	3.76	55.7	55.9	3.70 (3H, s)	3.83	56.8	56.4
MeO-4’	3.59 (3H, s)	3.62	55.7	55.1	3.61 (3H, s)	3.67	55.7	55.6	-		-		-		-	

*Piccinelli AL, Campo Fernandez M, Cuesta-Rubio O, Márquez Hernández I, De Simone F, Rastrelli L. Isoflavonoids isolated from Cuban *propolis*. Journal of Agricultural and Food Chemistry. 2005; 53(23): 9010-6. (Ref [11] of main manuscript)

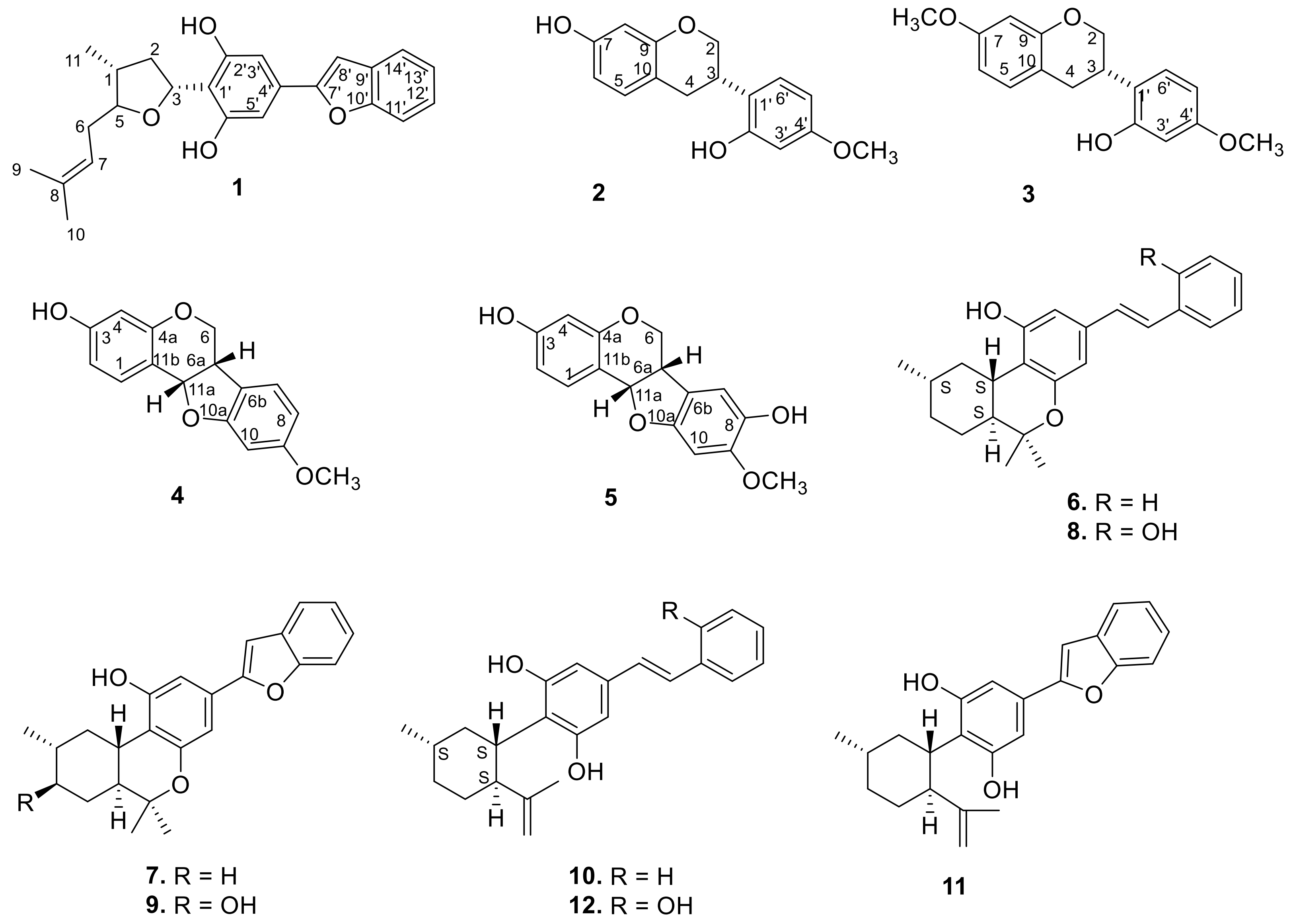

Figure S 1. Structures of isolated compounds **1-12**

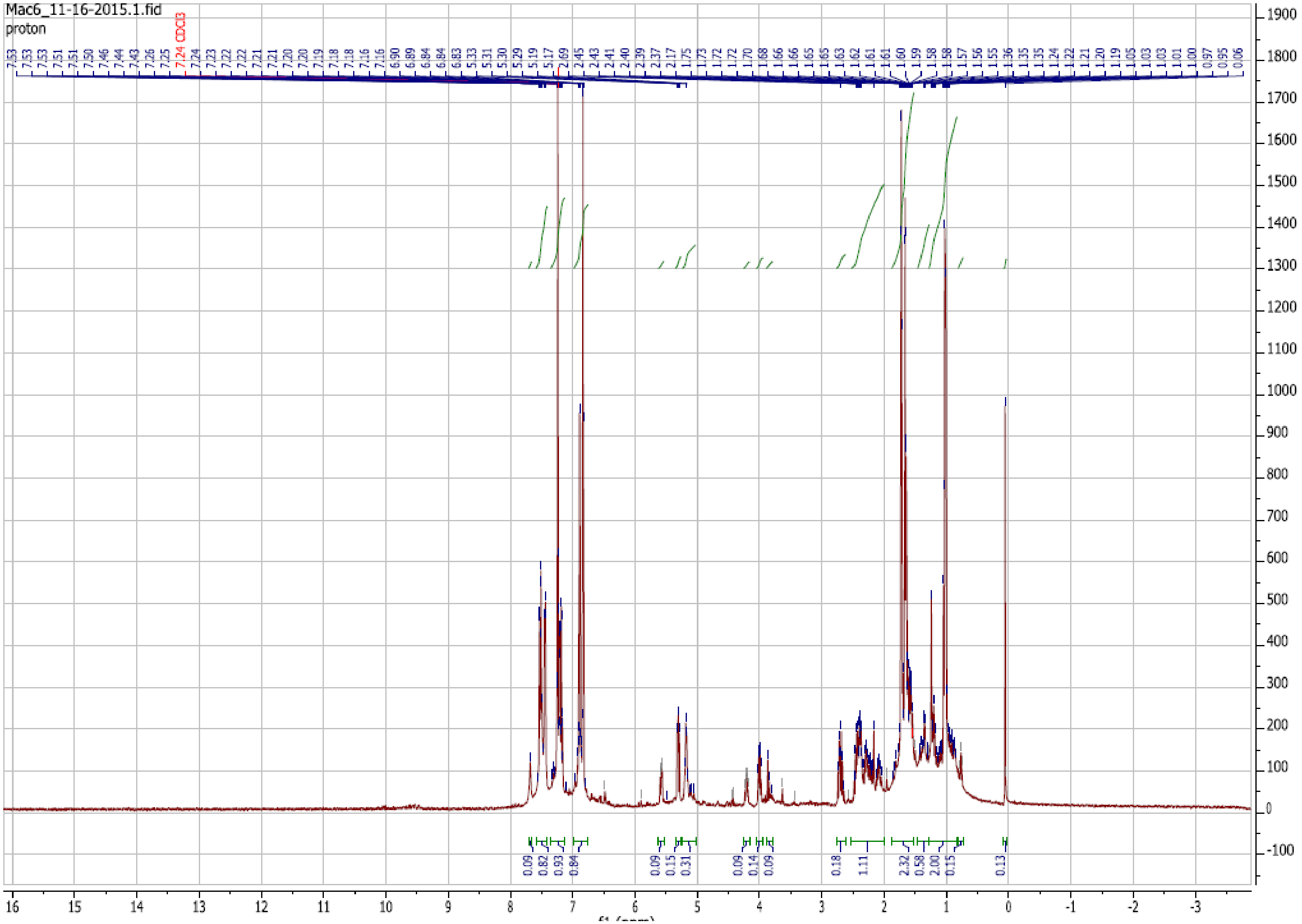

Figure S 2. . ^{1}H NMR spectrum of compound **1**.

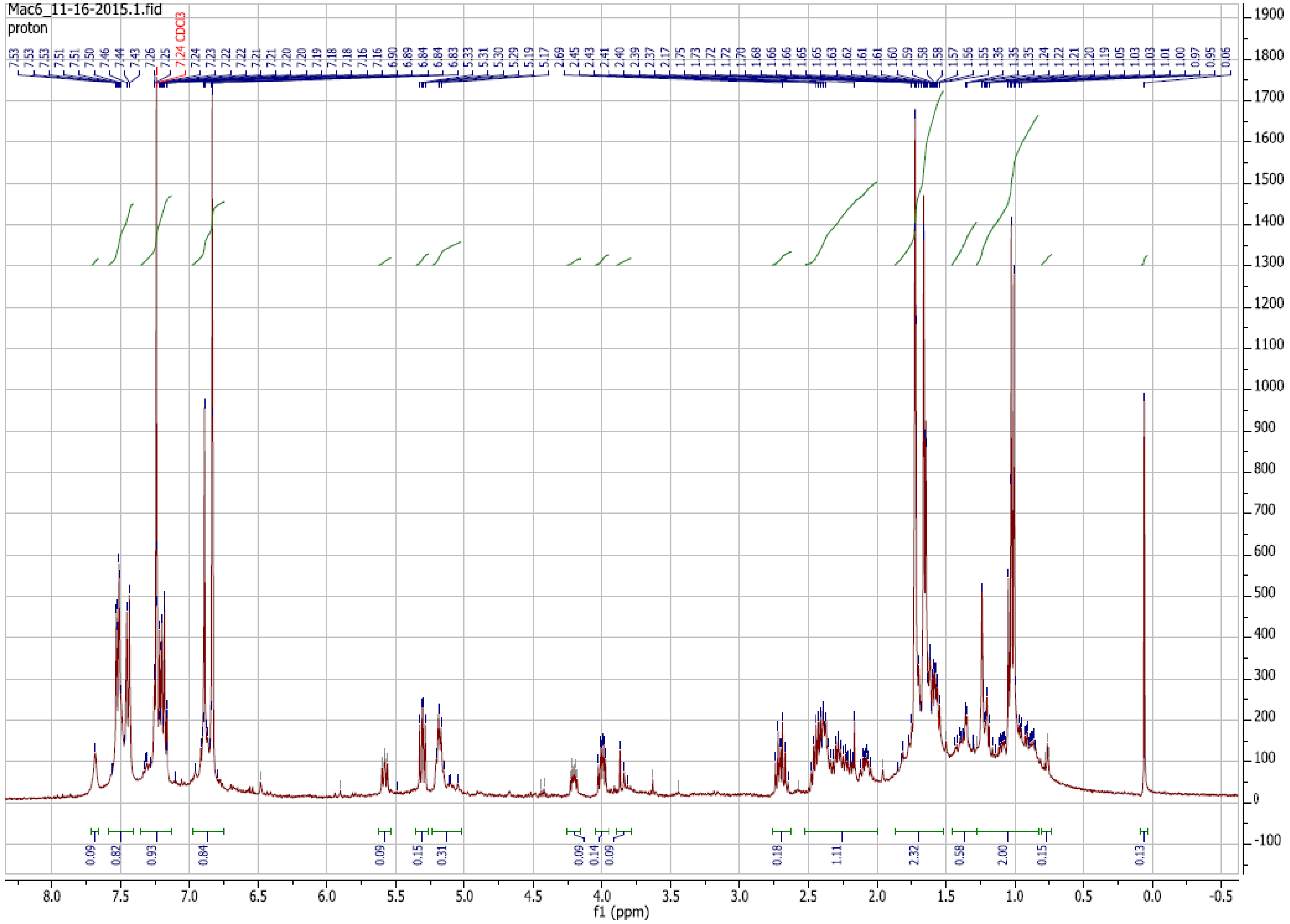

Figure S 3. ^{1}H NMR spectrum (expansion) of compound **1**.

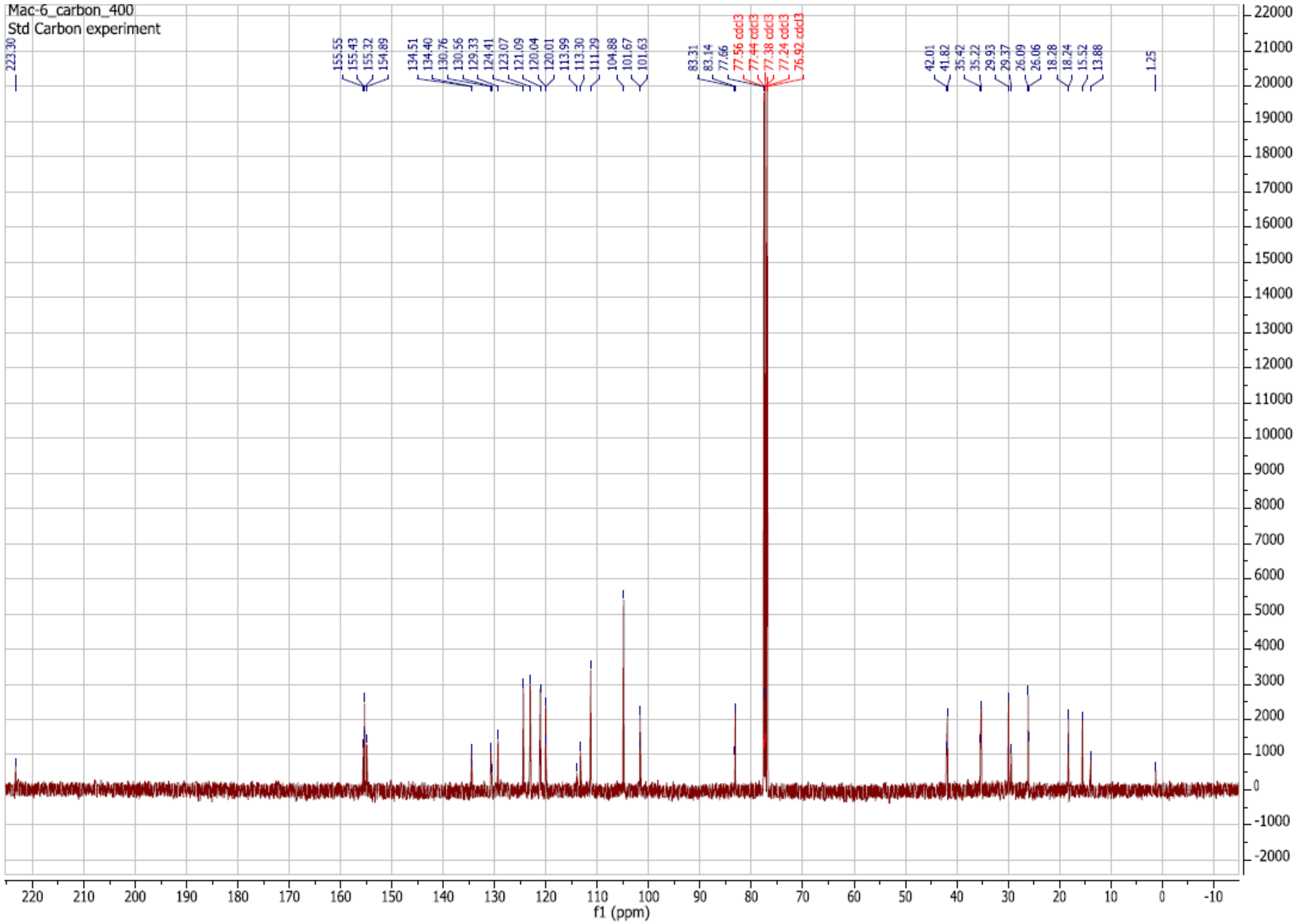

Figure S 4. ^{13}C NMR spectrum of compound **1**.

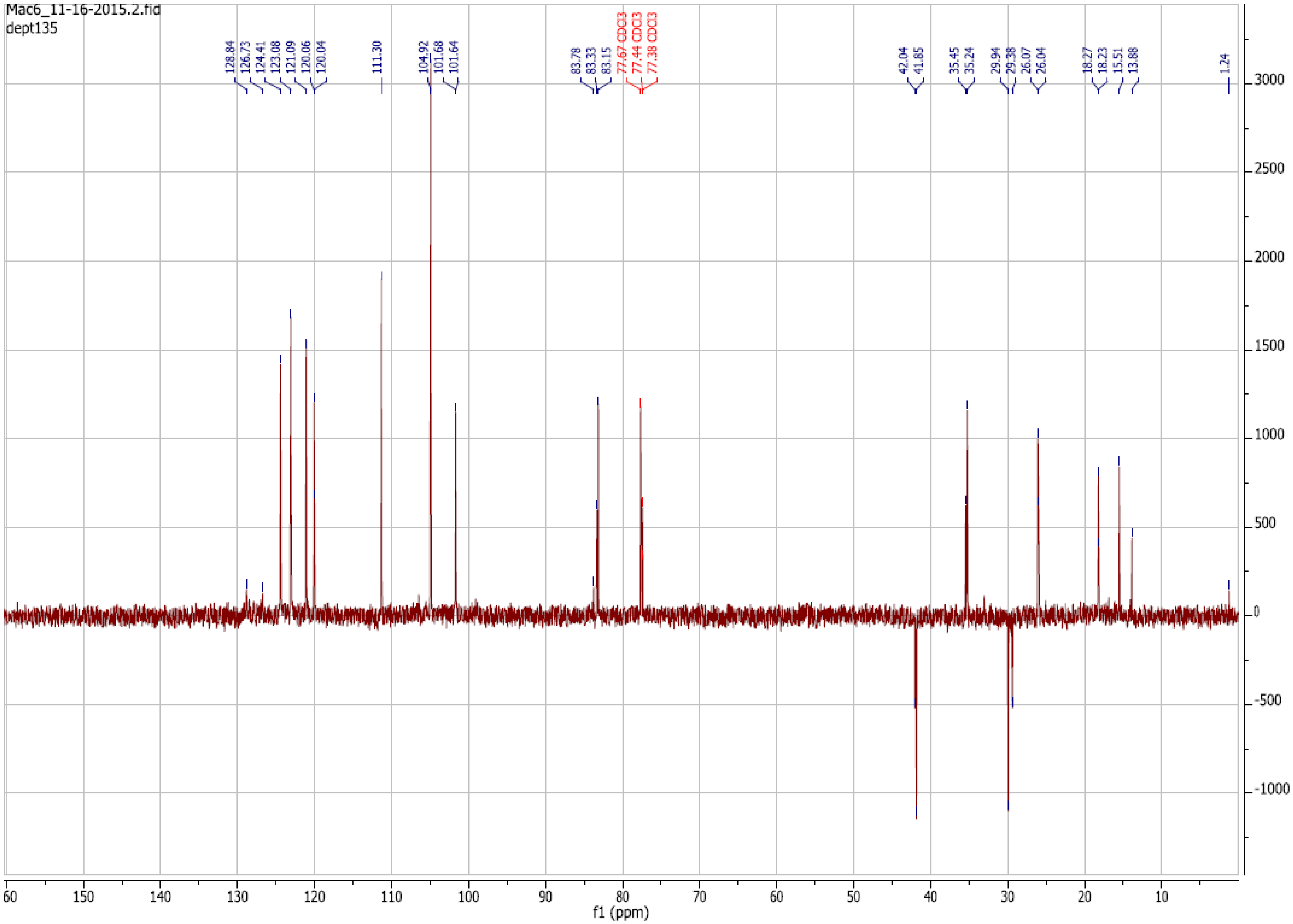

Figure S 5. . ^{13}C NMR Dept 135 spectrum of compound **1**.

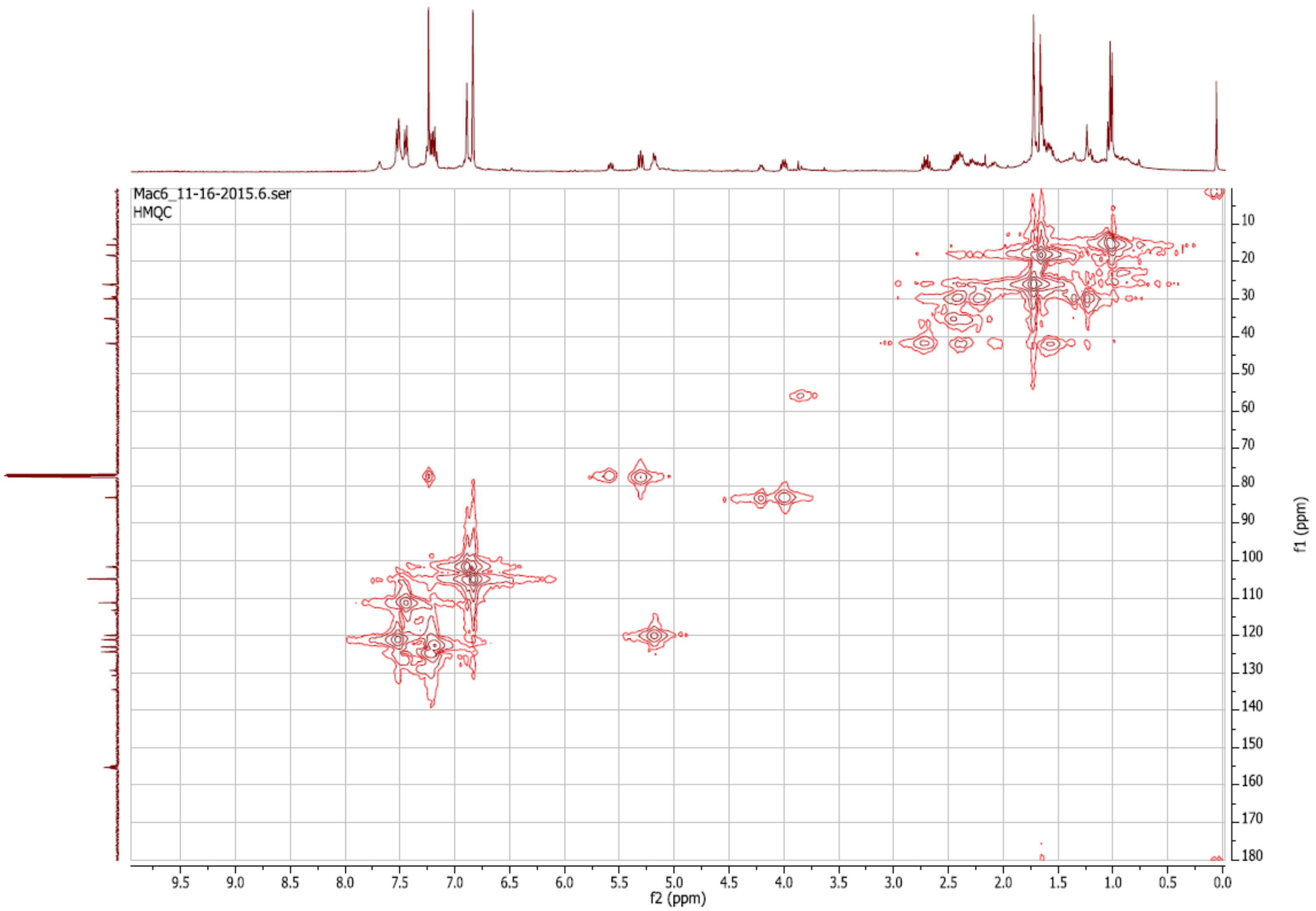

Figure S 6. . 2D NMR HMQC spectrum of compound **1**.

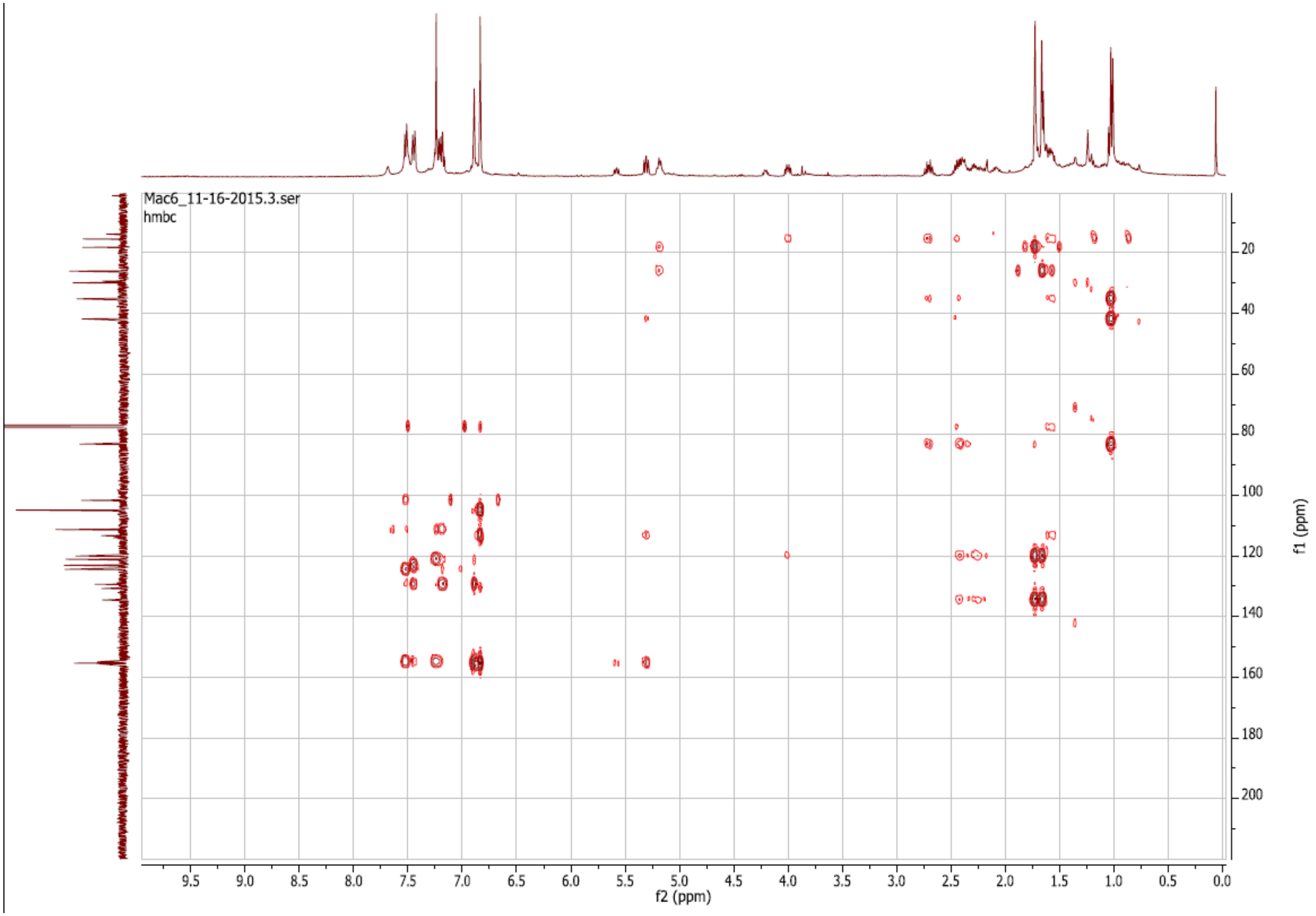

Figure S 7. . 2D NMR HMBC spectrum of compound **1**.

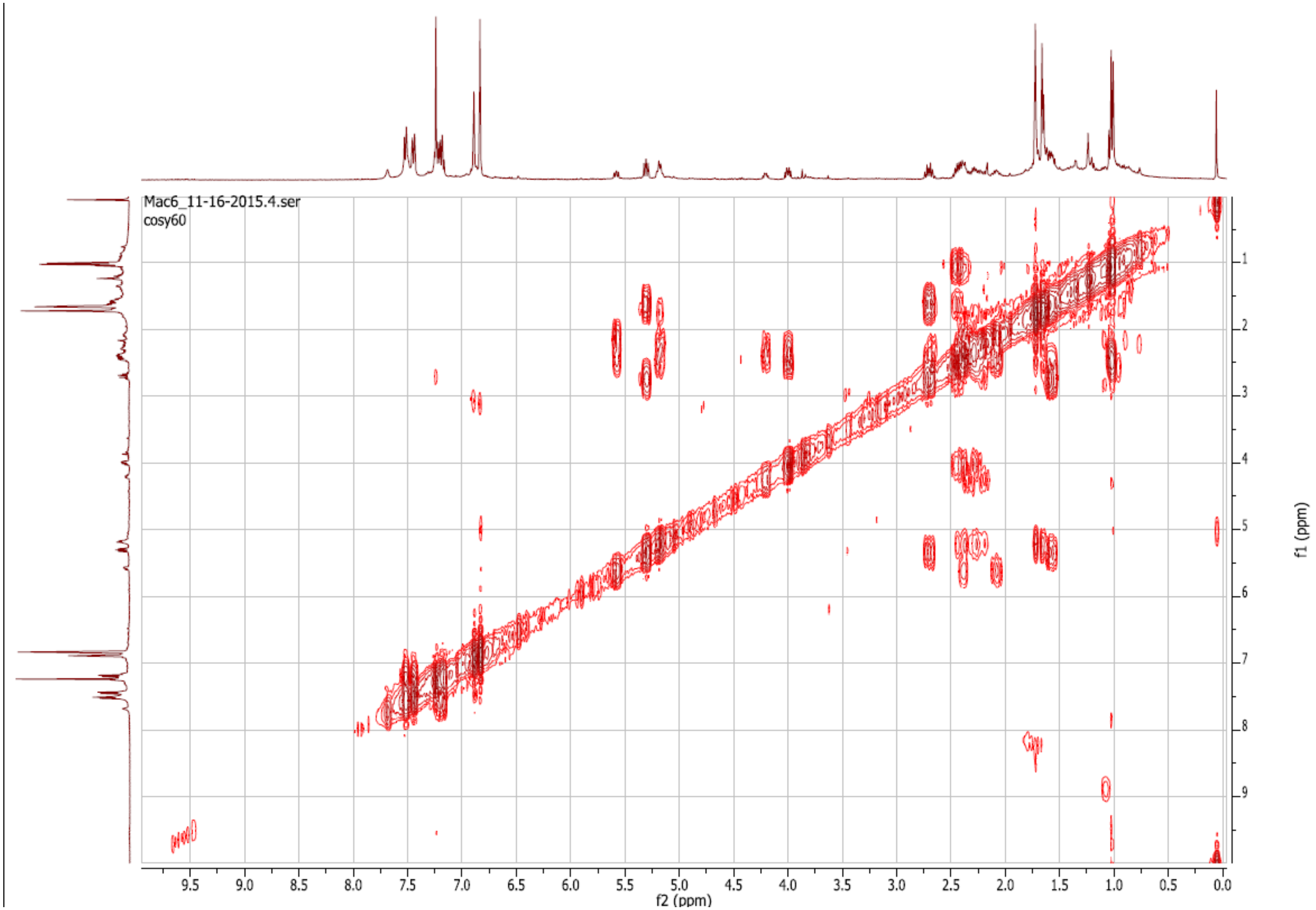

Figure S 8. . 2D NMR COSY spectrum of compound **1**.

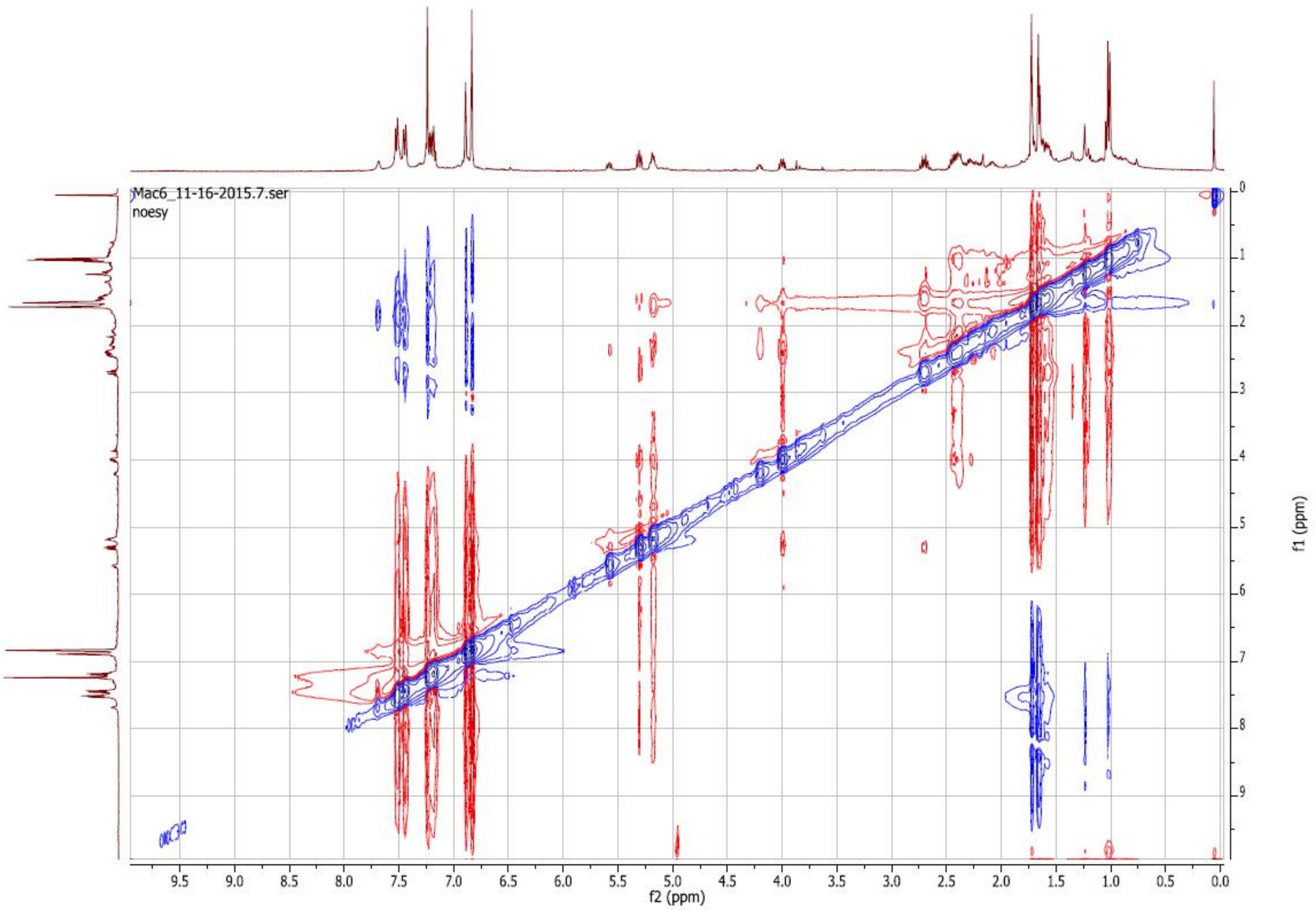

Figure S 9. 2D NMR NOESY spectrum of compound **1**.

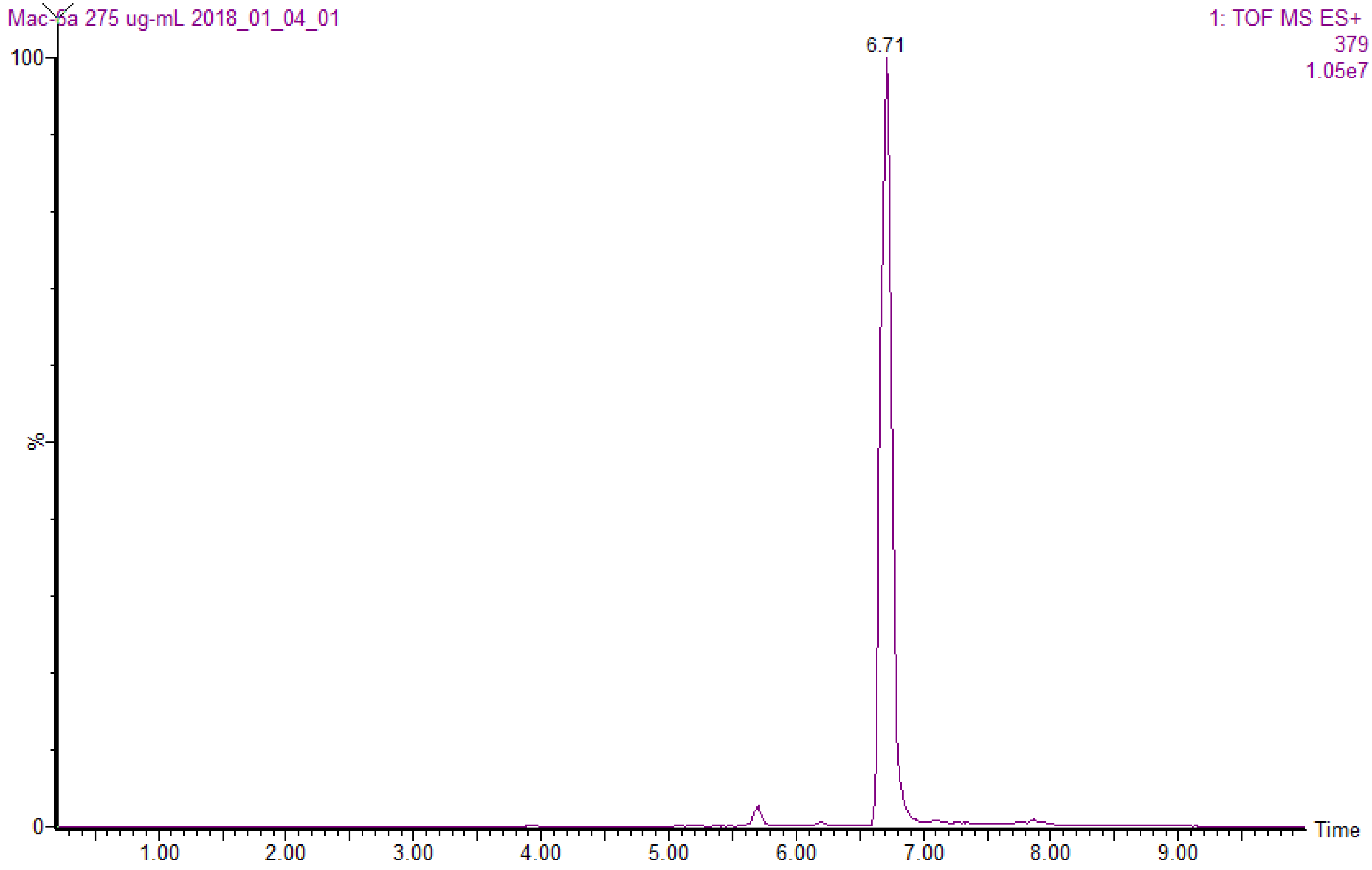

Figure S 10. . EIC (*m/z* = 379) chromatogram of compound **1**.

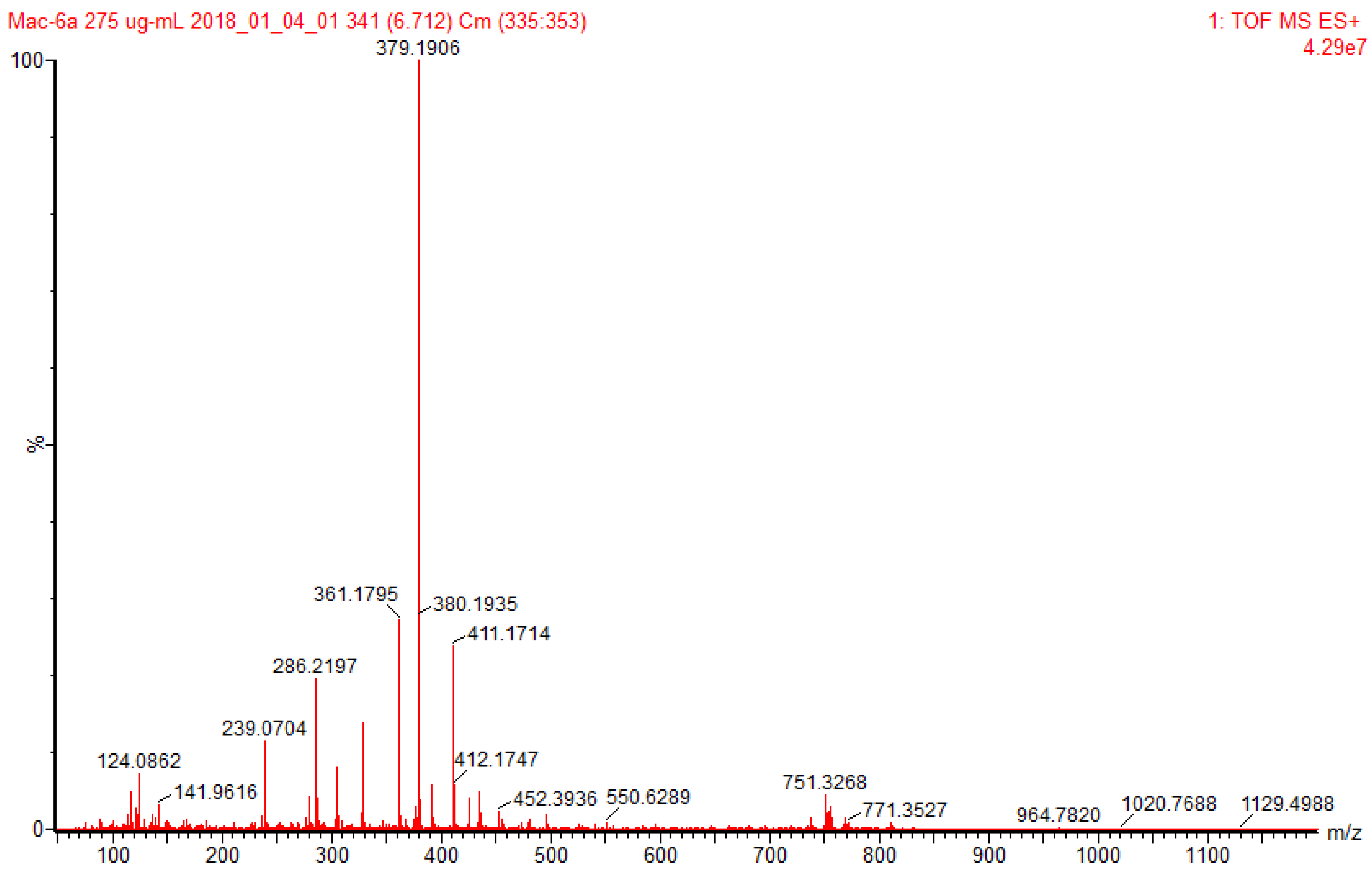

Figure S 11. HRMS spectrum of peak at 6.71 min of compound **1**.
